# Effect of Milk Protein and Whey Permeate in Large-Quantity Lipid-Based Nutrient Supplement on Early Child Development among Children with Stunting: A Randomized 2 × 2 Factorial Trial in Uganda

**DOI:** 10.3390/nu15122659

**Published:** 2023-06-07

**Authors:** Joseph Mbabazi, Hannah Pesu, Rolland Mutumba, Gareth McCray, Kim F. Michaelsen, Christian Ritz, Suzanne Filteau, André Briend, Ezekiel Mupere, Benedikte Grenov, Henrik Friis, Mette Frahm Olsen

**Affiliations:** 1 Department of Nutrition, Exercise and Sports, University of Copenhagen, 1958 Frederiksberg C, Denmark; mjosef2000@gmail.com (J.M.); hpe@nexs.ku.dk (H.P.); mutumbarolland@gmail.com (R.M.); kfm@nexs.ku.dk (K.F.M.); andre.briend@gmail.com (A.B.); bgr@nexs.ku.dk (B.G.); hfr@nexs.ku.dk (H.F.); 2 Department of Paediatrics and Child Health, Makerere University, Kampala P.O. Box 7072, Uganda; mupez@yahoo.com; 3 School of Medicine, Keele University, Keele ST5 5BG, UK; g.mccray@keele.ac.uk; 4 The National Institute of Public Health, University of Southern Denmark, 5230 Odense, Denmark; ritz@sdu.dk; 5 Faculty of Epidemiology and Population Health, London School of Hygiene and Tropical Medicine, London WC1E 7HT, UK; suzanne.filteau@lshtm.ac.uk; 6 Tampere Center for Child, Adolescent and Maternal Health Research, Faculty of Medicine and Health Technology, Tampere University, Tampere University Hospital, 33520 Tampere, Finland; 7 Department of Infectious Diseases, Rigshospitalet, 2100 Copenhagen, Denmark

**Keywords:** stunting, early child development, milk protein, whey permeate, LNS

## Abstract

Stunting affects 22% children globally, putting them at risk of adverse outcomes including delayed development. We investigated the effect of milk protein (MP) vs. soy and whey permeate (WP) vs. maltodextrin in large-quantity, lipid-based nutrient supplement (LNS), and LNS itself vs. no supplementation, on child development and head circumference among stunted children aged 1–5 years. We conducted a randomized, double-blind, community-based 2 × 2 factorial trial in Uganda (ISRCTN1309319). We randomized 600 children to one of four LNS formulations (~535 kcal/d), with or without MP (*n* = 299 vs. *n* = 301) or WP (*n* = 301 vs. *n* = 299), for 12 weeks or to no supplementation (*n* = 150). Child development was assessed using the Malawi Development Assessment Tool. Data were analyzed using linear mixed-effects models. Children had a median [interquartile range] age of 30 [23; 41] months and mean ± standard deviation height-for-age z-score of −3.02 ± 0.74. There were no interactions between MP and WP for any of the outcomes. There was no effect of either MP or WP on any developmental domain. Although LNS itself had no impact on development, it resulted in 0.07 (95%CI: 0.004; 0.14) cm higher head circumference. Neither dairy in LNS, nor LNS in itself, had an effect on development among already stunted children.

## 1. Introduction

Child stunting is one of the most important impediments to human development, globally affecting 22% of children under 5 years [1]. In the short-term, stunting places the child at an increased risk of morbidity and mortality, and is associated with lower cognitive and motor development [2]. In the long-term, it is associated with diminished growth and development, reduced productive capacity, and an increased risk of chronic diseases later in life [2].

In low- and middle-income countries (LMICs), 43% of children under 5 years are at risk of not reaching their developmental potential [3]. In sub-Saharan Africa, more than half of the children under five-years old experience developmental challenges, especially boys [4]. Notable identified risk factors for developmental delay are intrauterine growth restriction, stunting, iodine deficiency, anaemia, malaria, HIV, maternal depression and inadequate cognitive stimulation, while maternal schooling and breastfeeding, have been found to be protective [5].

As a notable predictor of development, head circumference is measured routinely especially in the first two years of life, a period during which the brain grows rapidly and the open sutures between the bones of the skull close. The increase in head circumference is more rapid at a younger age with up to 90% of the adult head size attained by two years. Head circumference increases by about 0.7 cm during the fifth year compared to 11 cm in the first year [6].

To improve early child development in line with the 2030 agenda for sustainable development [7], WHO recommends: (a) responsive caregiving, (b) promotion of early learning, (c) integration of caregiving and nutrition interventions, and (d) supporting maternal mental health [8]. The number of children with suspected developmental delay calls for multi-sectoral programs that incorporate most of the aforementioned factors. 

Studies examining the effect of small-quantity, lipid-based nutrient supplement (LNS) with complementary feeding on development have had mixed findings [9,10,11]. In a Cochrane review, compared to complementary feeding alone, a combination of LNS and complementary feeding mainly improved the milestone attainment “walking alone” [9]. There were generally no effects on other development domains and three of nine studies reported no effects on any of the assessed outcomes. Pre- and post-natal LNS supplementation has also shown conflicting results on child development [12], including effects on social-emotional score in Ghanaian children [11], while no effect was seen among Malawian children after 12 months supplementation [10]. One key limitation in previous studies may be the use of small-quantity LNS, which offers little energy (~100 kcal/d) and which may have failed to meet the children’s mineral and vitamin requirements. In addition, the protein content may have been both insufficient in quantity and in some cases of inferior quality to promote adequate brain development [13]. Milk protein has a high quality, and milk ingredients may support cognitive development in children [14] There is a paucity of literature regarding the effect of supplementation with large-quantity LNS on development.

Therefore, we aimed to measure the effect of milk protein (MP) vs. soy and whey permeate (WP) vs. maltodextrin, as part of large-quantity LNS (~535 kcal/d), as well as the effect of LNS itself vs. no supplementation on early child development among stunted children aged 1–5 years. We hypothesized that dairy in LNS, and/or LNS irrespective of dairy would affect child development.

## 2. Materials and Methods

### 2.1. Study Design

**Figure 1:**
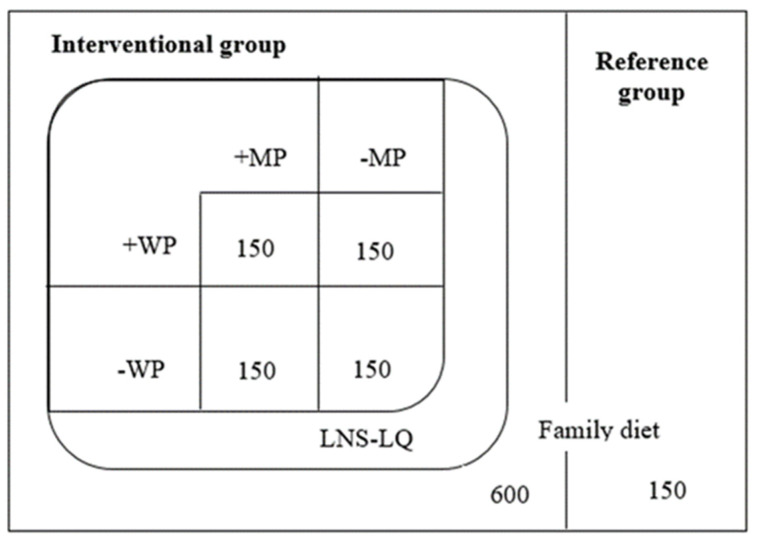
**Trial design**. The 2 × 2 factorial design showing the four different interventional products all large-quantity lipid-based nutrient supplements (LNS) containing either whey permeate (+WP) or no whey permeate (−WP). These were either added with milk protein (+MP) or no milk protein (−MP). Soy protein was added to those supplements with no MP while maltodextrin was added to those without WP. A fifth un-supplemented group was added as a reference.

This study reports secondary outcomes from the MAGNUS trial, a community-based randomized 2 × 2 factorial trial assessing the role of MP and WP in LNS on growth and development among stunted children (ISRCTN13093195). The primary outcome of the original trial was the effect of the intervention on height and knee-heel length as reported elsewhere [15,16]. The main outcome of the current study was child development. In the MAGNUS study, 750 children were randomized to one of four LNS formulations or no supplementation (Figure 1). Trial methods have been described in detail [15].

### 2.2. Study Participants

This study was conducted at two public health facilities in Walukuba division and Buwenge town council in Jinja district. The district is located about 75 km east of the capital city Kampala forming part of the ten districts in the Busoga sub-region in East-Central Uganda.

Study participants were identified from the surrounding villages and those found with height-for-age z-score (HAZ) < −2 and weight-for-height z-score (WHZ) > −3 were invited to the study clinic for eligibility screening. At the study clinic, children were enrolled if they met the following inclusion criteria: lived in the catchment area, caregiver willing to return for follow-up visits, and agreed to phone-follow-up plus home visits. Children who had severe acute malnutrition (WHZ < −3 or bilateral pitting oedema), medical complications that necessitated hospitalization, history of peanut or milk allergies, obvious disability impeding ability to eat or measure their length/height were excluded from the study. Children who were participating in another study, whose family planned to move away from the catchment area in the next six months, or had another child from the same household already included were excluded as well.

### 2.3. Randomization, Allocation Concealment and Blinding

As described previously [15], children were individually randomized to one of the four supplements (1:1:1:1) or to no supplementation (4:1). The study was block randomized with variable block sizes of 10 and 20, and stratified by site. The randomization sequences for each study site were generated using R by a staff member at the University of Copenhagen (UCPH), who was otherwise not involved in the study. The original sequence list was kept in a sealed envelope at UCPH and a hard copy of the site randomization list was provided to the study pharmacist in a sealed envelope. More details are provided elsewhere [15].

All investigators and outcome assessors were blinded with respect to LNS or no LNS, and to the ingredients contained in the differently coded LNS sachets. Caregivers and children were blinded with respect to the type of LNS allocated since all the four supplements were indistinguishable in appearance, smell and taste.

### 2.4. The Intervention

All caregivers, irrespective of study arm, received nutrition counselling at inclusion, in line with the national policy on infant and young child feeding [17]. Those randomized to the intervention arm received one LNS sachet of 100 g/day. The energy and macronutrient contents of the four LNS formulations were matched. Protein and carbohydrate constituted approximately 10% and 32% of the energy, respectively. Milk or soy protein constituted 50% of the total amount of protein, and WP or maltodextrin were added on top of a vitamin–mineral–mix in a peanut base. The LNS was manufactured and pre-packed by Nutriset (Malaunay, France) in coded zip-lock bags of 14 sachets. Each 100g LNS sachet provided ~535 kcal, which constituted up to half of the average daily energy requirements of a child and satisfied most of the daily micronutrient requirements (Supplementary Table S1). Those in the reference group received no supplement, and therefore continued with family diet only. The reference group received one 1 kg bar of laundry soap fortnightly. To discourage sharing, caregivers were informed that the LNS was only intended for the study child. In case of any siblings aged 6–59 months, the family received one extra ration of the same food supplement as the study child at each visit, irrespective of stunting status.

Compliance to LNS intake was measured by counting returned empty sachets and asking the caregiver how many sachets the child had consumed since the last visit. Missing sachets or data were all considered as lack of intake.

### 2.5. Study Visits

All caregivers returned fortnightly to collect LNS or laundry soap until the 12th week. Distribution took place in a closed room only after all other on-site study activities were completed for the participant. At baseline, data on the following were collected: demographic information, dietary intake including breastfeeding (defined as any breastfeeding in the previous 24 h), food insecurity assessment, anthropometry. Child development was assessed and blood samples collected for micronutrient and other clinical data analyses at baseline and endline. 

### 2.6. Sample Size Consideration

This was based on the primary outcome of the main trial (height and knee-heel length) where detecting 0.35 SD or a greater difference in any outcome between any of the two groups, at 5% significance level and 80% power, while allowing for 10% attrition, allowed us to recruit 150 children per group (600 in total based on the 4 combinations of MP and WP). If there were no interactions between the two experimental arms, then two groups of 300 children could be compared enabling differences of 0.24 SD to be detected.

### 2.7. Outcomes

Child development, the main outcome of the current study, was assessed using the Malawi development assessment tool (MDAT) version 6 [18] translated to both Lusoga and Luganda. The tool has been adapted and validated for use in LMICs and was developed in an African setting. It focuses on four domains including gross motor, fine motor, language, and social skills with 39, 42, 40 and 36 milestones in each domain, respectively. The MDAT is primarily an observation-based tool with standardized items assessed by trained research assistants whom we refer to as child development officers (CDOs). During assessments, most of the items were observed while some, mainly in the social domain, were assessed based on caregiver recall. Normal age-specific reference values for each domain were used as a starting point to select appropriate items when testing each child. The CDO first performed a forward test until the child failed six consecutive items, thereby marking the rest of the items above as failed. If the child passed six consecutive items in the forward test, all items below were marked passed; if not, a backward test was done until six consecutive items were passed. As a quality check, an independent concurrent assessment was done routinely (after every 20 developmental assessments per CDO) by another CDO using the MDAT to compare results. In case of any discrepancy, consensus was arrived by referring to the standard operating procedure and soliciting views from CDOs regarding that particular item.

Head circumference (mm) was measured in triplicate using a windowed, nonelastic paper-head circumference tape (SECA 212, Hamburg, Germany), in line with accepted international standards [19]. This was taken around the widest possible circumference of the child’s head at 2-finger widths (using the first and middle figures) above the eyebrows on the forehead, and the occiput. This was performed via replacement during measurements, aided by the caregiver holding the tape securely in place at the occipital bun.

### 2.8. Additional Data

The child and caregiver’s participation during the MDAT assessment was observed by the CDO. This was evaluated based on an adapted version of the Behaviour Observation Inventory from the Bayley Scales of Infant and Toddler Development [20]. The child was assessed on how happy, engaged, cooperative and anxious they appeared during the majority of the assessment, and how much support their caregiver provided without necessarily influencing the test.

The child’s developmental stimulation at home was assessed using an African validated family care indicators (FCI) questionnaire [21]. The caregiver was asked about the level of stimulation at home in four subscales, including availability of reading materials and their number, source of play materials, variety of play materials, and engagement with an adult family member (15+ years old) in various interactive activities. These interactive activities included reading books, telling stories, singing songs, taking the child out, playing with the child, and counting or drawing for the child.

All anthropometric measurements were repeated in triplicate and the median used. Weight was obtained using an electronic scale with a double weighing function (Seca 876, Hamburg, Germany). Length (measured in children < 24 months) and height (measured in children 24 months and older) was obtained using a wooden Shorrboard (Weigh and measure LLC, Olney, MD, USA) ensuring five points of contact with repositioning between measurements. Mid-upper arm circumference (MUAC) was measured using a non-elastic MUAC tape (UNICEF SD, Copenhagen, Denmark). 

All caregivers received nutrition counselling using the national guidelines on infant and young child feeding (IYCF) [17]. Demographic information was recorded and full clinical examination of the child was performed. Dietary intake was assessed by 24 h dietary recall using an interviewer-administered questionnaire. Food security was calculated based on the USAID household, food-insecurity access scale [22], while dietary diversity was calculated based on the WHO global nutrition-monitoring framework operational guidance [23].

Venous blood was drawn from each child, transported to the field laboratory, processed, temporarily stored at −20 °C before being transported to Kampala for storage at −80 °C. Processed samples were later transferred to Denmark and Germany in dry ice for analysis of the acute phase proteins using a sandwich enzyme-linked immunosorbent assay (VitminLab, Willstaett, Germany).

### 2.9. Statistical Analyses

Paper case report forms were used to collect data, which were then doubly entered in EpiData with inbuilt range checks before uploading to REDCap (Open-Source Vanderbilt University) to a secure server at UCPH weekly. Data comparison was performed and any discrepancies were verified by both entrants with the aid of the hard-copy case report forms and consultations from respective research assistants before rectification. Statistical analysis was performed using Stata SE14 and descriptive statistics presented as mean ± SD, median [IQR], or % (n).

We generated internal MDAT developmental age-adjusted z-scores. This involved using the 2-parameter logistic (2PL) model [24] and generalized additive model for location, scale and shape (GAMLSS) [25]. An item-response theory (IRT) analysis was conducted using unidimensional 2-parameter-logistic (2PL) models in the R package MIRT [26] to create child-specific development scores for each time point for the provided MDAT item responses, for both overall (total score) and domain specific. Thereafter, a GAMLSS was utilized, in the R package [25] to generate an age contingent measure of ability based upon the development scores from IRT model, hence removing the impact of age on development.

Statistical analyses were based on the intention-to-treat (ITT) principle using available case data. We analyzed the 2 × 2 factorial design using linear mixed effects models in all analyses, utilizing the ‘xtmixed’ command in Stata version 14. We first tested for any interactions between the two interventions in the matrix (MP and WP). In case of no interaction effect, we then tested for the main effects on child development. The compound symmetry variance–covariance structure was assumed, and no robust standard errors were applied because the linear mixed model fits showed no marked departures from the model assumptions. Furthermore, we compared the intervention (LNS) irrespective of milk ingredients among 600 children against the 150 un-supplemented group. Prespecified subgroup analyses were performed for all outcomes, testing for any effect modifications by sex, breastfeeding, stunting severity, inflammation (serum α-1 acid glycoprotein, AGP ≥ 1.2 g/L), and stimulative home environment (having any children’s book at home, with >2 sources of play materials of >3 varieties, and with >3 engagements with older family members in interactive activities).

All linear mixed models were adjusted for the baseline value of the outcome, age, sex, and season, as fixed effects, as well as site, as a random effect. Restricted maximum likelihood estimation was used, and model assumptions were assessed visually using residual and Q–Q plots. Estimates, and 95% CI, were reported, and a significance level of 0.05 was used.

A per protocol analysis was performed, retaining only those children who remained until the study-endline visit, and had at least 80% compliance with LNS supplementation for those in the interventional group. Details on the measure of compliance are reported in the main paper [16].

### 2.10. Ethics

This study was approved by the Makerere University School of Medicine Research and Ethics Committee (#REC REF 2019-013) and the Uganda National Council of Science and Technology (SS 4927). We also obtained consultative approval from the Danish National Committee on Biomedical Research Ethics (1906848). All study staff took courses in Good Clinical Practice and Human Subject Protection prior to study start. We obtained oral and written informed consent in Lusoga, Luganda or English from all participants’ caregivers. Illiterate caregivers were taken through the process in the presence of a literate witness. All caregivers consented after their understanding of the information was double checked by a different study or facility staff member, using an informed consent questionnaire.

## 3. Results

### 3.1. Participant Characteristics 

**Figure 2:**
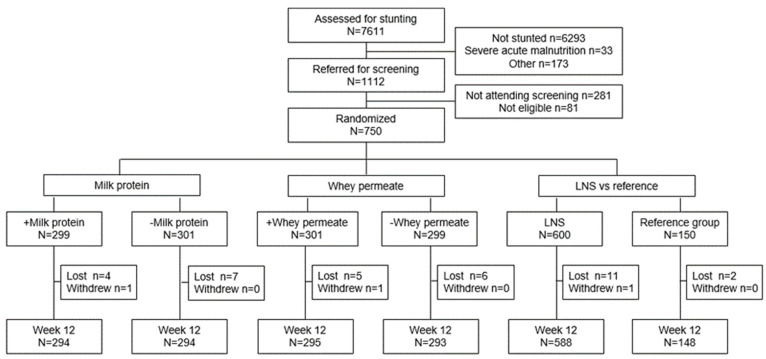
Trial profile.

Between February and September 2020, we screened 7611 children at various village sites. Of these, 1193 were stunted, 750 of whom were enrolled and randomized into one of five groups (Figure 2), and 736 (98.1%) of the children completed the 12-week follow-up.

At inclusion, the median [interquartile range, IQR] age was 30 [23; 41] months, 45% (338) were girls, and the mean ± SD HAZ was −3.02 ± 0.74. The mean ±SD MDAT Z-scores for gross motor, fine motor, language, social and total MDAT scores were −0.19 ± 1.02, −0.13 ± 1.04, −0.15 ± 1.03, −0.13 ± 1.03, and −0.20 ± 1.00, respectively. Generally, the randomization resulted in baseline equivalence with respect to potential confounders (Table 1).

### 3.2. Development among the Unsupplemented Children

Over the 12-week follow-up period, there were increments in developmental scores across all domains among the un-supplemented children (Table 2). Additionally, their head circumference increased by 0.28 (95%CI: 0.21; 0.35) cm on average. The observed estimates were only attenuated with further adjustments for potential confounders, except for fine motor domain (Supplementary Table S2).

### 3.3. Main Effect of Milk Ingredients and/or LNS on Development

The primary intention-to-treat analysis assessed the effects of MP and WP based on the factorial design among the 600 children who received LNS. There was no interaction between MP and WP for any of the outcomes (Table 3). Therefore, the results are presented as the main effect of each of the experimental milk ingredients. Neither MP nor WP had an effect on any of the outcomes. Furthermore, LNS irrespective of milk ingredients had no effect on any developmental domain but it resulted in 0.07 (95%CI: 0.004; 0.14) cm higher head circumference (Table 3). Similar estimates were observed without adjustments for age, sex, and season and with further adjustments for no maternal schooling, positive malaria test, and current breastfeeding (Supplementary Table S3). 

### 3.4. Subgroup Effect of Milk Ingredients, and LNS in Itself, on Development

We assessed whether the effects of MP and WP were modified by sex, breastfeeding, stunting severity, baseline inflammation, and a stimulative home environment. The effect of MP on social skills was modified by sex and breastfeeding (interaction, *p* = 0.02), due to a larger effect of MP among girls compared to boys, and a lower effect of MP in breastfed compared to non-breastfed (Supplementary Table S4). Accordingly, MP increased social skills by 0.22 (95%CI: 0.01; 0.44) Z-scores in girls, and reduced social skills in those that are breastfed by −0.39 (95%CI: −0.79; 0.01), although the latter was only marginally significant (Supplementary Table S4).

The effect of MP on cognition was modified by a stimulative home environment but with marginal significance. This was due to a larger effect of MP on fine motor (interaction, *p* = 0.07) and on language skills (interaction, *p* = 0.06) among those with a stimulative compared to non-stimulative home environment (Supplementary Table S4).

Whey permeate was associated with lower motor and social skills among children with severe compared to moderate stunting. This corresponded to 0.07 (95%CI: −0.15; 0.29) and 0.22 (95%CI: −0.01; 0.44) lower gain in gross and fine motor scores, respectively, among severely compared to moderately stunted children (Supplementary Table S5). The corresponding effect on social skills was −0.02 (95%CI: −0.25; 0.20) with marginal significance. The effect of LNS was modified by breastfeeding and inflammation. Irrespective of milk ingredient, LNS interacted with breastfeeding resulting in 0.42 (95%CI: −0.08; 0.92), 0.39 (95%CI: −0.10; 0.88) and 0.48 (95%CI: −0.02; 0.99) higher increment in gross motor, language and social skill scores, respectively, among those breastfed compared to non-breastfed children (Supplementary Table S6). Additionally, inflammation modified the effect of LNS resulting in a larger effect on gross motor (0.28, 95%CI: −0.08; 0.65) and language skills (0.24, 95%CI: −0.12; 0.59) among those children with, compared to without, inflammation at baseline (Supplementary Table S6). 

The adherence to the nutritional intervention was reported elsewhere [16]. Of those randomized to supplementation (*n* = 600), 86% of the caregivers returned >80% empty sachets. Our results from the intention-to-treat analysis were retained after a per protocol analysis (Supplementary Table S7). 

## 4. Discussion

In our nutrition intervention study using large-quantity LNS, neither MP nor WP had an effect on any development domain or head circumference. Irrespective of milk ingredients, LNS vs. no supplementation had no effect on any development domain but seemed to increase head circumference. A further test for interactions revealed that the effect of MP on social skills was modified by sex and current breastfeeding. The effect of WP on motor and social skills was modified by stunting severity while the effect of LNS irrespective of dairy was modified by current breastfeeding and inflammation.

Several nutrition studies have shown that milk protein, or milk per se [27,28], is associated with increased head and brain size but with some inconsistences among different age groups and for other functional outcomes. Irrespective of milk, supplemented children gained an additional 0.07 cm head circumference compared to their un-supplemented counterparts. This was moreover among children with stunting aged ~32 months on average, followed up for only 12 weeks.

Despite the scant literature on the developmental effects of supplementation with large-quantity LNS, studies using small-quantity LNS have also had inconsistent results [9,10,11]. In a Cochrane review [9], out of the nine studies that reported data on psychomotor and neurodevelopmental outcomes, four studies reported early milestone attainment (walking alone), two reported better motor development, and similar to our findings, three reported no impact of small-quantity LNS plus complementary feeding vs. complementary feeding alone. In that review, compared to micronutrient powders, small-quantity LNS with complementary feeding was associated with improved problem solving abilities, while one study had comparable developmental outcomes among children given small-quantity LNS plus complementary feeding and those given fortified blended flours [9]. In an earlier meta-analysis, pre- and post-natal nutrition interventions were associated with only small effects on mental development [12] in LMICs, with improved personal-social skills among Ghanian children [11], and no effect among 6–18 months old Malawian children [10].

Among other challenges, comparability is difficult across studies due to variations in definitions and measurements used for the different variables. Additionally, most previous studies have used small-quantity LNS, which provides minimal energy (~100 kcal/d), to meet the part of the child’s daily energy requirements (~1000 kcal/d). 

To some extent, our results concur with these studies that LNS intervention does not generally result in development, despite using large-quantity supplements providing more energy (~535 kcal/day), and fully satisfying the child’s daily micronutrient requirements. We already reported that the same milk ingredients had no effect, but rather, LNS, irrespective of milk ingredients, benefited growth [16]. Of note, milk protein was compared against soy protein isolate which is a high-quality protein with a low content of antinutrients.

In the management of malnutrition, there were concerns about the polyunsaturated fat acid (PUFA) composition of earlier WHO standard LNS versions in facilitating neurocognitive and immune recovery. This was mainly due to the content of linoleic acid in those products when given at high doses (150–200 kcal/kg/day), thus, calls for data on optimal omega-6 and omega-3 PUFA content. Studies testing novel products revealed that adding omega-3 DHA or a reduction of omega-6 linoleic acid by using high-oleic peanuts improved overall omega-3 (DHA) status with subsequent development [29,30]. Increasing omega-3 α-linolenic acid alone in the product showed no impact. Compared to our study, intake from LNS was less than the case for RUTF, and we used LNS that contained moderate levels of α-linolenic acid. An earlier study by our team among Burkinabe children with moderate acute malnutrition (MAM) tested the effectiveness of LNS or corn-soy blend (CSB), with either soy isolate or dehulled soy, and either 0%, 20%, or 50% of milk protein aided in child development [31]. The study found children to show a greater improvement in language scores if children received supplements with milk (20%: 0.09 [−0.01, 0.19], *p* = 0.08 and 50%: 0.11 [0.01, 0.21], *p* = 0.02), with statistical significance observed for 50% milk [31]. Fine motor scores were also improved among children receiving milk after the addition of 20% milk to CSB (0.18 [0.03, 0.33], *p* = 0.02). Compared to our study, these studies were among different populations (SAM and MAM children) and used somewhat different food supplements.

A stimulative home environment may have interacted with MP, resulting in more rapid ontogenetic development, hence, a higher increase in cognitive scores among those with stimulative compared to non-stimulative home environment. It may have also been more of an interaction with the household’s socioeconomic status (SES), consequently resulting in better cognitive development. This is a key research gap noted by some scholars as to whether it is lack of stimulation or poverty that is responsible for poor development [32,33]. A study in the United Kingdom examined a chaotic home environment as a potential mediator between parental SES and cognitive development [34]. Direct relations between parental SES and cognitive ability were partly mediated by the home atmosphere by up to 16% for change in cognition. A chaotic home environment may lead to a series of biological effects that could potentially compromise health and child’s developmental potential [35]. Notably, children in our study from households with a single income earner had low developmental scores at baseline. Moreover, those that hailed from households with a single income earner and no maternal schooling had a non-stimulative home environment. Thus, this indicates more of a mediation effect between stimulative home environment and SES rather than MP *per se* on cognitive development.

Studies have reported lower early child development scores among stunted than non-stunted children [36]. These studies also found maternal schooling and body mass index to be a predictor of development pointing towards the SES—child development interplay. Premised on such a background, our interaction findings, between WP and stunting severity leading to a higher increase in motor and social skills among moderate vs. severe stunting, may not be due to WP per se. This is partly due to the fact that WP provides greater amounts of minerals compared to empty calories by maltodextrin. 

From the literature, breastfeeding has been associated with improved cognitive performance and socio-affective response [37]. In our findings on interactions by breastfeeding and LNS irrespective of milk ingredients, there was higher increase in cognition and social skills among breastfed vs. non-breastfed children. This is partly due to the fact that compared to non-breastfed, breastfed children had lower baseline total Z-scores, i.e., −0.54 ± 1.00 vs. −0.15 ± 0.99 between breastfed vs. non-breastfed (*p* = 0.0004). During the 12-week follow-up, these could have been attributed more to the developmental benefits of breastfeeding [37] than the rest. This is in addition to the reverse causality between breastfeeding and lower developmental scores that we established among these children at baseline. Conversely, children that were inflamed at baseline (serum AGP ≥ 1.2 g/L) tended to have lower developmental scores than the uninflamed, i.e., −0.27 ± 0.95 vs. −0.07 ± 1.06 total Z-score, respectively (*p* = 0.01). This could partly explain the observed interaction as children are prone to have scored low at baseline due to being inflamed, i.e., feeling sick, and over the course of the intervention, they recovered, returning to normal performance, hence performing better at endline as evidenced by higher motor and language scores. 

Key strengths of our study are the randomized 2 × 2 factorial design with an un-supplemented group for comparison, a very low attrition rate, use of a contextually developed and local tool to measure the outcome and pre-specified subgroup analysis plan to avoid chance findings. Limitations may include the incomplete blinding of the caregivers with respect to supplementation, despite measures in place to curb this, recall, and information bias from caregiver’s reports on the family care indicators.

## 5. Conclusions

We did not find any effects of milk protein vs. soy protein, or whey permeate vs. maltodextrin as part of LNS, or LNS in itself vs. no supplementation, on early child development among stunted children. Additionally, dairy did not have an effect on head circumference, but irrespective of dairy, LNS increased head circumference in these children.

Milk protein tends to improve cognitive skills more among children from stimulative compared to non-stimulative homes, possibly as a result of interaction with their SES. Conversely, the interaction between WP and stunting severity resulting in increased motor and social skills development, among the moderately, compared to severely stunted, is likely a result of an additional dose–response effect with severity on top of the established stunting—poor child development association. Irrespective of milk ingredients, the effect modifier via breastfeeding on LNS onto development, supports the continued promotion of breastfeeding for improved growth and development among children.

## Figures and Tables

**Table 1:** Baseline characteristics among stunted children randomized to a lipid-based nutrient supplement (LNS) with milk or soy protein and whey permeate or maltodextrin (*n* = 4 × 150 = 600), or no supplement (*n* = 150) for 12 weeks ^1^.

Baseline Characteristic	LNS with Milk Protein (*n* = 299)	LNS with Soy Protein (*n* = 301)	LNS with Whey Permeate (*n* = 301)	LNS with Maltodextrin (*n* = 299)	LNS (*n* = 600)	No Supplement (*n* = 150)
**Sociodemographic data**						
Age (months)	31 [24; 41]	29 [22; 40]	31 [23; 42]	28 [23; 39]	30 [23; 41]	32 [23; 41]
Sex (male)	52.2% (156)	57.1% (172)	52.8% (159)	56.5% (169)	54.7% (328)	56.0% (84)
Currently breastfed	14.1% (42)	12.7% (38)	16.1% (48)	10.7% (32)	13.4% (80)	10.1% (15)
Residence (rural)	55.0% (160)	55.8% (163)	56.5% (166)	54.3% (157)	55.4% (323)	53.1% (78)
Household size	5 [4; 7]	5 [4; 6]	5 [4; 7]	5 [4; 6]	5 [4; 7]	5 [4; 7]
**Socioeconomic status**						
Single income earner	69.9% (209)	71.33 (214)	69.8% (210)	71.5% (213)	70.6% (423)	68.7% (103)
Income spent on food (>50%)	69.2% (207)	67.1% (202)	70.4% (212)	65.9% (197)	68.2% (409)	69.3% (104)
No maternal schooling	48.4% (138)	47.7% (137)	50.2% (146)	45.9% (129)	48.1% (275)	44.7% (63)
Severe food insecurity ^2^	66.8% (189)	65.5% (188)	68.3% (198)	63.9% (179)	66.1% (377)	68.7% (101)
Diverse diet ^3^	26.8% (80)	26.0% (78)	26.1% (78)	26.8% (80)	26.4% (158)	25.7% (38)
Female-headed households	23.0% (68)	19.9% (59)	19.9% (59)	23.0% (68)	21.5% (127)	20.6% (30)
**Anthropometric data**						
Weight (kg)	10.7 ± 1.9	10.5 ± 2.0	10.6 ± 2.0	10.5 ± 1.9	10.6 ± 2.0	10.6 ± 2.1
Height [19] ^4,5^	81.9 ± 7.1	81.3 ± 7.5	81.9 ± 7.6	81.4 ± 7.1	81.6 ± 7.3	81.9 ± 7.5
Head circumference [19] ^5^	47.3 ± 1.8	47.1 ± 1.7	47.2 ± 1.7	47.2 ± 1.8	47.2 ± 1.8	47.3 ± 1.9
Height-for-age z-score	−3.02 ± 0.74	−3.04 ± 0.73	−3.06 ± 0.75	−2.99 ± 0.72	−3.03 ±0.73	−2.99 ± 0.75
Weight-for-height z-score	−0.27 ± 1.03	−0.42 ± 0.93	−0.34 ± 0.95	−0.35 ± 1.01	−0.35 ± 0.98	−0.43 ± 1.03
Weight-for-age z-score	−1.87 ± 0.91	−1.97 ± 0.79	−1.94 ± 0.84	−1.90 ± 0.87	−1.92 ± 0.86	−1.97 ± 0.83
**Micronutrient and clinical data**						
Malaria (RDT positive)	39.9% (117)	36.6% (108)	40.2% (119)	36.3% (106)	38.3% (225)	44.9% (67)
Haemoglobin < 110 g/L	64.9% (192)	64.6% (192)	64.3% (191)	65.2% (193)	64.8% (384)	63.3% (95)
Serum CRP > 10 mg/L	20.8% (61)	24.6% (73)	21.6% (64)	23.7% (70)	22.7% (134)	19.3% (29)
Serum AGP ≥ 1.2 g/L	62.3% (185)	63.6% (471)	65.5% (194)	63.4% (187)	64.5% (381)	60.0% (90)
**Family care indicators**						
Any children’s book at home	33.8% (101)	33.2% (100)	32.9% (99)	34.1% (102)	33.5% (201)	32.0% (48)
Sources of play materials (>2) ^6^	18.4% (55)	19.3% (58)	18.3% (55)	19.4% (58)	18.8% (113)	19.3% (29)
Variety of play materials (>3) ^6^	34.5% (103)	29.9% (90)	36.2% (109)	28.1% (84)	32.2% (193)	28.0% (42)
Family interaction (>3) ^6^	32.4% (97)	36.2% (109)	35.2% (106)	33.4% (100)	34.3% (206)	30.7% (46)

^1^ Values are mean ± standard deviation, % (frequency, n), and median [interquartile range], ^2^ calculated based on the HFIAS (Access) score by USAID, FANTA project v.3 on a 1–4 scale ranging from food secure—severely food insecure, ^3^ calculated based on MDDS, eating from 5 or more of 8 food groups including breastmilk in the past 24 h, ^4^ children under 24 months were measured lying, ^5^ and was reported according to the ICMJE guidelines [19], ^6^ based on an earlier established scale just above the average for these children. **Abbreviations**; HFIAS: household food insecurity access scale, MDDS: minimum dietary diversity score, RDT: rapid diagnostic test, CRP: creatine reactive protein, AGP: α-1-acid glycoprotein, MDAT: Malawi development assessment tool, ICMJE: International committee of medical journal editors.

**Table 2:** Early child development among the 148 stunted children randomized to no supplementation and completed 12 weeks follow-up ^1^.

Outcome	Baseline (t = 0)	Endline (t = 12 Week)	Difference	
	Mean ± SD	Mean ± SD	β (95% CI)	*p* Value
**MDAT domains (Z-scores)**				
Gross motor	−0.21 ± 1.03	0.22 ± 1.01	0.43 (0.22; 0.63)	<0.001
Fine motor	−0.24 ± 1.05	0.17 ± 0.89	0.41 (0.19; 0.62)	<0.001
Language	−0.16 ± 1.10	0.22 ± 0.85	0.38 (0.18; 0.58)	<0.001
Social skills	−0.19 ± 0.96	0.16 ± 0.99	0.35 (0.16; 0.54)	<0.001
Total score	−0.23 ± 1.03	0.26 ± 0.92	0.49 (0.30; 0.68)	<0.001
**Other outcomes**				
Head circumference [19]	47.33 ± 1.95	47.61 ± 1.88	0.28 (0.21; 0.35)	<0.001

^1^ Data of mean ± standard deviation at both timepoints, difference β (95% confidence interval), and *p*-value based on paired *t* test.

**Table 3:** Intention-to-treat analysis: Effect of milk protein and whey permeate in a lipid-based nutrient supplement (LNS) on early child development among 750 children with stunting. Analysis based on the 2 × 2 factorial design among the 600 supplemented and comparison of 600 supplemented vs. 150 un-supplemented stunted children ^1^.

Outcomes		Milk vs. Soy Protein (*n* = 299 vs. *n* = 301)		Whey Permeate vs. Maltodextrin (*n* = 301 vs. *n* = 299)		LNS vs. No Supplement (*n* = 600 vs. *n* = 150)	
	Interaction, *p*	B (95% CI)	*p*	B (95% CI)	*p*	B (95% CI)	*p*
**MDAT domains (Z-scores)**							
Gross motor	0.20	−0.05 (−0.19; 0.09)	0.48	0.09 (−0.06; 0.23)	0.23	−0.05 (−0.22; 0.11)	0.51
Fine motor	0.95	−0.04 (−0.19; 0.11)	0.61	−0.01 (−0.16; 0.14)	0.86	−0.07 (−0.24; 0.09)	0.39
Language	0.92	−0.03 (−0.18; 0.11)	0.64	−0.06 (−0.21; 0.08)	0.40	−0.08 (−0.24; 0.08)	0.31
Social skills	0.42	0.04 (−0.11; 0.18)	0.62	0.12 (−0.02; 0.27)	0.09	−0.05 (−0.21; 0.12)	0.58
Total score	0.95	−0.03 (−0.17; 0.11)	0.66	0.01 (−0.14; 0.15)	0.92	−0.09 (−0.25; 0.07)	0.29
**Other outcomes**							
Head circumference [19]	0.17	0.02 (−0.04; 0.08)	0.59	−0.04 (−0.10; 0.02)	0.21	0.07 (0.004; 0.14)	0.04

^1^ Data are p for interaction between milk protein and whey permeate, and main effect B (95% confidence interval) and *p*-value of each intervention based on linear mixed effect models adjusted for baseline value of the outcome, age, sex, season, and site.

## Data Availability

The Ugandan act on Data Protection and Privacy and the European act on General Data Protection Regulation do not allow for personal data to be made available to other researchers without prior written approval from relevant institutions and authorities. For further information, please contact the corresponding author.

## Supplementary Materials

The following supporting information can be downloaded at: https://www.mdpi.com/article/10.3390/nu15122659/s1, Table S1: Supplement composition of each large quantity lipid-based nutrient supplement (LNS); Table S2: Sensitivity analysis; Early child development among 148 stunted children randomized to no supplementation and completed the 12-weeks follow-up; Table S3: Sensitivity analysis; Effect of milk protein and whey permeate in lipid-based nutrient supplement (LNS) on early child development among 750 children with stunting. Intention-to-treat analysis based on the 2 × 2 factorial design among the 600 supplemented and comparison of 600 supplemented vs. 150 un-supplemented stunted children ^1^; Table S4: Subgroup effects of milk protein in a lipid-based nutrient supplement (LNS) on early child development; Table S5: Subgroup effects of whey permeate in a lipid-based nutrient supplement (LNS) on child development; Table S6: Subgroup effects of lipid-based nutrient supplement (LNS) on child development; Table S7: Per protocol analysis; Effect of Milk protein and whey permeate in a lipid-based nutrient supplement (LNS) on child development among 662 children with stunting.
