# Supplementary material for: Effect of Milk Protein and Whey Permeate in Large-Quantity Lipid-Based Nutrient Supplement on Early Child Development among Children with Stunting: A Randomized 2 × 2 Factorial Trial in Uganda
Source: Nutrients. 2023 Jun 7;15(12):2659. doi: 10.3390/nu15122659 (PMC10301406; doi:10.3390/nu15122659)
Supplement: Supplementary file 1 [file nutrients-15-02659-s001.zip › nutrients-2394555-supplementary.docx]

**Supplementary tables**

| Table S.1: Supplement composition of each large-quantity lipid-based nutrient supplement (LNS) | | | | | |
| --- | --- | --- | --- | --- | --- |
|  |  | **Milk protein**^1^  **Whey permeate** | **Milk protein**^1^  **Maltodextrin** | **Soy protein**^2^  **Whey permeate** | **Soy protein**^2^  **Maltodextrin** |
|  |  |  |  |  |  |
| Protein quality | DIAAS^3^ |  |  |  |  |
| 6-35 months |  | 0·93 | 0·93 | 0·78 | 0·78 |
| 36+ months |  | 1·10 | 1·10 | 0·93 | 0·93 |
| Nutrient | **Per 100 g** |  |  |  |  |
| Macronutrients |  |  |  |  |  |
| Energy | kcal | 531 | 535 | 530 | 534 |
| Carbohydrates | g | 42 | 43 | 42 | 43 |
| Lactose | g | 15·7 | 0·4 | 15·3 | 0 |
| Proteins | g | 13·9 | 13·5 | 13·9 | 13·5 |
| Milk | g | 7·15 | 6·75 | 0·40 | 0 |
| Vegetable | g | 6·75 | 6·75 | 13·50 | 13·50 |
| Lipids | g | 33·7 | 33·7 | 33·7 | 33·7 |
| Linoleic acid C18:2 | g | 3·0 | 3·0 | 3·0 | 3·0 |
| Linoleic acid C18:3 | g | 0·5 | 0·5 | 0·5 | 0·5 |
| Minerals |  |  |  |  |  |
| Calcium | mg | 691 | 594 | 691 | 594 |
| Copper | mg | 1·65 | 1·65 | 1·65 | 1·65 |
| Iron | mg | 12 | 12 | 12 | 12 |
| Iodine | mg | 127 | 113 | 127 | 113 |
| Magnesium | mg | 199·2 | 175·8 | 199·2 | 175·8 |
| Manganese | mg | 1·8 | 1·8 | 1·8 | 1·8 |
| Phosphorous | mg | 661 | 539 | 661 | 539 |
| Potassium | mg | 1315 | 985 | 1315 | 985 |
| Sodium | mg | 84 | 7 | 156 | 79 |
| Selenium | µg | 30 | 30 | 30 | 30 |
| Zinc | mg | 12·5 | 12·5 | 12·5 | 12·5 |
| Vitamins^4^ |  |  |  |  |  |
| Vitamin A | µg | 619 | 619 | 619 | 619 |
| Vitamin B1 | mg | 1·2 | 1·1 | 1·2 | 1·1 |
| Vitamin B12 | µg | 3·2 | 3·0 | 3·2 | 3·0 |
| Vitamin B2 | mg | 3·1 | 2·8 | 2·7 | 2·4 |
| Niacin | mg | 14·9 | 14·6 | 14·9 | 14·6 |
| Pantothenic acid | mg | 5·7 | 4·5 | 5·7 | 4·5 |
| Vitamin B6 | mg | 2·1 | 2·0 | 2·1 | 2·0 |
| Biotin | µg | 74·1 | 67·6 | 74·1 | 67·6 |
| Folic acid | µg | 223 | 223 | 223 | 223 |
| Vitamin C | mg | 67·9 | 67·6 | 67·9 | 67·6 |
| Vitamin D | µg | 16·9 | 16·9 | 16·9 | 16·9 |
| Vitamin E | mg | 18 | 18 | 18 | 18 |
| Vitamin K | µg | 30 | 30 | 30 | 30 |
| ^1^ Milk protein isolate (casein and whey) ^2^ Soy protein isolate. ^3^ Digestible indispensable amino acid score. ^4^ Target values by the end of the product’s shelf life. | | | | | |

| Table S2: Sensitivity analysis; Early child development among 148 stunted children randomized to no supplementation and completed the 12-weeks follow-up ^1^ | | | | |
| --- | --- | --- | --- | --- |
| Outcome | Baseline (t=0) | Endline (t=12week) | Difference^2^ | |
|  | **Mean ±SD** | **Mean ±SD** | **β (95% CI)** | **P value** |
| **MDAT domains (Z-scores)** |  |  |  |  |
| Gross motor | -0.21 ±1.03 | 0.22 ±1.01 | 0.16 (-0.01; 0.32) | 0.067 |
| Fine motor | -0.24 ±1.05 | 0.17 ±0.89 | 0.05 (-0.09; 0.20) | 0.482 |
| Language | -0.16 ±1.10 | 0.22 ±0.85 | 0.16 (0.04; 0.29) | 0.009 |
| Social skills | -0.19 ±0.96 | 0.16 ±0.99 | 0.28 (0.10; 0.45) | 0.002 |
| Total score | -0.23 ±1.03 | 0.26 ±0.92 | 0.18 (0.04; 0.33) | 0.011 |
| **Other outcomes** |  |  |  |  |
| Head circumference [1] | 47.33 ±1.95 | 47.61 ±1.88 | 0.10 (0.94; 1.05) | <0.001 |
|  |  |  |  |  |
| ^1^Data of mean ±standard deviation at both timepoints, Difference^2^ β (95% confidence interval), and p-value based on linear regression while adjusting for baseline value of the outcome, age, sex, no maternal schooling, malaria, and current breastfeeding. | | | | |

| Table S3: Sensitivity analysis; Effect of milk protein and whey permeate in lipid-based nutrient supplement (LNS) on early child development among 750 children with stunting. Intention-to-treat analysis based on the 2 × 2 factorial design among the 600 supplemented and comparison of 600 supplemented vs. 150 un-supplemented stunted children ^1^ | | | | | | | | | | | | | |
| --- | --- | --- | --- | --- | --- | --- | --- | --- | --- | --- | --- | --- | --- |
| **Outcomes** |  | **Milk vs soy protein**  **(n=299 vs n=301)** | | | | **Whey permeate vs maltodextrin**  **(n=301 vs n=299)** | | | | **LNS vs no supplement**  **(n=600 vs n=150)** | | | |
|  | **Interaction, P** | Unadjusted B (95% CI) | P | Adjusted B (95% CI) | P | Unadjusted B (95% CI) | P | Adjusted B (95% CI) | P | Unadjusted B (95% CI) | P | Adjusted B (95% CI) | P |
| **MDAT domains (Z-scores)** |  |  |  |  |  |  |  |  |  |  |  |  |  |
| Gross motor | 0.20 | -0.06 (-0.20; 0.09) | 0.442 | -0.02 (-0.17; 0.12) | 0.778 | 0.07 (-0.07; 0.22) | 0.317 | 0.09 (-0.06; 0.24) | 0.229 | -0.05 (-0.22; 0.11) | 0.514 | -0.03 (-0.20; 0.14) | 0.734 |
| Fine motor | 0.95 | -0.05 (-0.20; 0.10) | 0.542 | -0.02 (-0.16; 0.13) | 0.836 | -0.03 (-0.18; 0.12) | 0.725 | -0.01 (-0.16; 0.13) | 0.867 | -0.07 (-0.24; 0.09) | 0.397 | -0.05 (-0.22; 0.11) | 0.520 |
| Language | 0.92 | -0.04 (-0.19; 0.11) | 0.580 | -0.02 (-0.17; 0.13) | 0.823 | -0.08 (-0.23; 0.07) | 0.280 | -0.10 (-0.25; 0.05) | 0.186 | -0.08 (-0.24; 0.08) | 0.335 | -0.09 (-0.25; 0.08) | 0.296 |
| Social skills | 0.42 | 0.03 (-0.11; 0.18) | 0.672 | 0.03 (-0.12; 0.18) | 0.706 | 0.12 (-0.03; 0.26) | 0.119 | 0.11 (-0.05; 0.26) | 0.171 | -0.04 (-0.21; 0.12) | 0.608 | -0.04 (-0.20; 0.13) | 0.676 |
| Total score | 0.95 | -0.04 (-0.19; 0.10) | 0.557 | -0.02 (-0.16; 0.13) | 0.811 | -0.01 (-0.16; 0.13) | 0.849 | -0.02 (-0.16; 0.13) | 0.791 | -0.08 (-0.25; 0.08) | 0.307 | -0.06 (-0.22; 0.10) | 0.441 |
| **Other outcomes** |  |  |  |  |  |  |  |  |  |  |  |  |  |
| Head circumference [1] | 0.17 | 0.01 (-0.06; 0.07) | 0.825 | 0.03 (-0.03; 0.09) | 0.369 | -0.05 (-0.12; 0.01) | 0.120 | -0.03 (-0.09; 0.03) | 0.370 | 0.07 (-0.00; 0.15) | 0.050 | 0.06 (-0.01; 0.14) | 0.074 |
| ^1^Data are p for interaction between milk protein and whey permeate, and main effect B (95% confidence interval) and p-value of each intervention based on linear mixed effect models with both the unadjusted and values adjusted for baseline value of the outcome, age, sex, season, no maternal schooling, malaria, current breastfeeding, and site. | | | | | | | | | | | | | |

| Table S.4: Subgroup effects of milk protein in lipid-based nutrient supplement (LNS) on early child development by sex, breastfeeding status, stunting severity, inflammation, and stimulative home environment among the children with stunting who received LNS (n=600) | | | | | | | | | | |
| --- | --- | --- | --- | --- | --- | --- | --- | --- | --- | --- |
| Outcome | **Sex** | | **Breastfeeding** | | **Severe stunting** | | **Inflammation** | | **Stimulative home environment** | |
|  | Boy | | Not breastfeeding | | Moderate stunting | | No inflammation | | non-stimulative | |
|  | Girl | | Breastfeeding | | Severe stunting | | Inflammation | | Stimulative | |
|  | **Int, P** | **B (95% CI)** | **Int, P** | **B (95% CI)** | **Int, P** | **B (95% CI)** | **Int, P** | **B (95% CI)** | **Int, P** | **B (95% CI)** |
| Gross motor | 0.81 | -0.07 (-0.26; 0.13) | 0.34 | -0.08 (-0.24; 0.07) | 0.61 | -0.02 (-0.21; 0.17) | 0.41 | -0.02 (-0.18; 0.15) | 0.94 | -0.13 (-0.38; 0.12) |
|  |  | -0.03 (-0.25; 0.18) |  | 0.12 (-0.27; 0.52) |  | -0.10 (-0.32; 0.12) |  | -0.17 (-0.47; 0.14) |  | -0.16 (-0.94; 0.61) |
| Fine motor | 0.79 | -0.06 (-0.26; 0.14) | 0.13 | 0.01 (-0.15; 0.17) | 0.34 | -0.10 (-0.30; 0.09) | 0.75 | -0.05 (-0.22; 0.12) | 0.07 | -0.13 (-0.37; 0.11) |
|  |  | -0.02 (-0.24; 0.20) |  | -0.33 (-0.73; 0.08) |  | 0.04 (-0.18; 0.27) |  | 0.01 (-0.31; 0.32) |  | 0.60 (-0.14; 1.35) |
| Language | 0.17 | 0.06 (-0.14; 0.26) | 0.79 | -0.03 (-0.19; 0.13) | 0.11 | -0.14 (-0.33; 0.05) | 0.72 | -0.04 (-0.21; 0.13) | 0.06 | -0.08 (-0.33; 0.16) |
|  |  | -0.15 (-0.36; 0.07) |  | -0.09 (-0.49; 0.32) |  | 0.10 (-0.12; 0.32) |  | 0.03 (-0.28; 0.34) |  | 0.69 (-0.06; 1.46) |
| Social skills | 0.02 | -0.12 (-0.31; 0.08) | 0.02 | 0.10 (-0.05; 0.26) | 0.61 | 0.07 (-0.12; 0.26) | 0.44 | -0.003 (-0.17; 0.16) | 0.80 | 0.005 (-0.24; 0.25) |
|  |  | 0.22 (0.01; 0.44) |  | -0.39 (-0.79; 0.01) |  | -0.01 (-0.23; 0.21) |  | 0.14 (-0.18; 0.45) |  | -0.10 (-0.87; 0.67) |
| Total score | 0.74 | -0.01 (-0.20; 0.18) | 0.52 | -0.02 (-0.17; 0.14) | 0.30 | -0.10 (-0.29; 0.09) | 0.79 | -0.04 (-0.20; 0.12) | 0.14 | -0.10 (-0.32; 0.13) |
|  |  | -0.06 (-0.27; 0.15) |  | -0.15 (-0.55; 0.24) |  | 0.05 (-0.17; 0.27) |  | 0.01 (-0.30; 0.31) |  | 0.47 (-0.24; 1.18) |
| Head circumference [1] | 0.72 | 0.01 (-0.08; 0.09) | 0.04 | -0.004 (-0.07; 0.06) | 0.85 | 0.02 (-0.06; 0.10) | 0.59 | 0.01 (-0.06; 0.08) | 0.19 | -0.06 (-0.16; 0.04) |
|  |  | 0.03 (-0.06; 0.12) |  | 0.19 (0.02; 0.35) |  | 0.01 (-0.08; 0.10) |  | 0.05 (-0.08; 0.18) |  | 0.16 (-0.15; 0.47) |
| Data are p for interaction and stratum-specific estimates (95% confidence interval) based on linear mixed effect models adjusted for baseline value, age, sex, season, and site  Abbreviations; Int: Interaction, CI: confidence interval | | | | | | | | | | |

| Table S.5: Subgroup effects of whey permeate in lipid-based nutrient supplement (LNS) on early child development by sex, breastfeeding status, stunting severity, inflammation, and stimulative home environment among the children with stunting who received LNS (n=600) | | | | | | | | | | |
| --- | --- | --- | --- | --- | --- | --- | --- | --- | --- | --- |
| Outcome | **Sex** | | **Breastfeeding** | | **Severe stunting** | | **Inflammation** | | **Stimulative home environment** | |
|  | Boy | | Not breastfeeding | | Moderate stunting | | No inflammation | | non-stimulative | |
|  | Girl | | Breastfeeding | | Severe stunting | | Inflammation | | Stimulative | |
|  | **Int, P** | **B (95% CI)** | **Int, P** | **B (95% CI)** | **Int, P** | **B (95% CI)** | **Int, P** | **B (95% CI)** | **Int, P** | **B (95% CI)** |
| Gross motor | 0.72 | 0.11 (-0.08; 0.31) | 0.24 | 0.05 (-0.10; 0.21) | 0.045 | 0.23 (0.04; 0.41) | 0.67 | 0.11 (-0.06; 0.27) | 0.68 | 0.07 (-0.18; 0.31) |
|  |  | 0.06 (-0.16; 0.27) |  | 0.31 (-0.09; 0.72) |  | -0.07 (-0.29; 0.15) |  | 0.03 (-0.27; 0.34) |  | -0.10 (-0.87; 0.66) |
| Fine motor | 0.11 | 0.10 (-0.10; 0.30) | 0.31 | -0.03 (-0.19; 0.13) | 0.02 | 0.15 (-0.05; 0.34) | 0.98 | -0.004 (-0.17; 0.17) | 0.77 | -0.09 (-0.33; 0.15) |
|  |  | -0.15 (-0.37; 0.07) |  | 0.21 (-0.21; 0.62) |  | -0.22 (-0.44; 0.01) |  | -0.001 (-0.33; 0.31) |  | 0.03 (-0.72; 0.78) |
| Language | 0.22 | 0.02 (-0.18; 0.22) | 0.45 | -0.09 (-0.25; 0.07) | 0.30 | 0.02 (-0.17; 0.21) | 0.55 | -0.08 (-0.25; 0.09) | 0.20 | -0.07 (-0.31; 0.17) |
|  |  | -0.16 (-0.38; 0.05) |  | 0.08 (-0.34; 0.49) |  | -0.13 (-0.36; 0.09) |  | 0.03 (-0.29; 0.34) |  | 0.45 (-0.31; 1.21) |
| Social skills | 0.15 | 0.22 (0.02; 0.42) | 0.89 | 0.12 (-0.04; 0.28) | 0.07 | 0.25 (0.05; 0.44) | 0.61 | 0.15 (-0.02; 0.32) | 0.63 | -0.03 (-0.27; 0.22) |
|  |  | 0.01 (-0.21; 0.22) |  | 0.09 (-0.32; 0.50) |  | -0.02 (-0.25; 0.20) |  | 0.06 (-0.25; 0.37) |  | -0.23 (-0.99; 0.54) |
| Total score | 0.14 | 0.11 (-0.09; 0.29) | 0.75 | -0.004 (-0.16; 0.15) | 0.03 | 0.16 (-0.03; 0.35) | 0.67 | -0.002 (-0.17; 0.16) | 0.59 | -0.05 (-0.28; 0.17) |
|  |  | -0.11 (-0.32; 0.10) |  | 0.07 (-0.34; 0.47) |  | -0.17 (-0.38; 0.05) |  | 0.07 (-0.23; 0.38) |  | 0.15 (-0.56; 0.87) |
| Head circumference [1] | 0.17 | -0.00 (-0.08; 0.08) | 0.48 | -0.04 (-0.11; 0.02) | 0.43 | -0.02 (-0.10; 0.06) | 0.43 | -0.03 (-0.10; 0.04) | 0.84 | -0.05 (-0.15; 0.05) |
|  |  | -0.09 (-0.18; 0.00) |  | 0.02 (-0.15; 0.20) |  | -0.07 (-0.17; 0.02) |  | -0.09 (-0.22; 0.04) |  | -0.08 (-0.39; 0.23) |
| Data are p for interaction and stratum-specific estimates (95% confidence interval) based on linear mixed effect models adjusted for baseline value, age, sex, season, and site  Abbreviations; Int: Interaction, CI: confidence interval | | | | | | | | | | |

| Table S.6: Subgroup effects of lipid-based nutrient supplement (LNS) on early child development by sex, breastfeeding status, stunting severity, inflammation, and stimulative home environment among the children with stunting who received LNS (n=600) | | | | | | | | | | |
| --- | --- | --- | --- | --- | --- | --- | --- | --- | --- | --- |
| Outcome | **Sex** | | **Breastfeeding** | | **Severe stunting** | | **Inflammation** | | **Stimulative home environment** | |
|  | Boy | | Not breastfeeding | | Moderate stunting | | No inflammation | | non-stimulative | |
|  | Girl | | Breastfeeding | | Severe stunting | | Inflammation | | Stimulative | |
|  | **Int, P** | **B (95% CI)** | **Int, P** | **B (95% CI)** | **Int, P** | **B (95% CI)** | **Int, P** | **B (95% CI)** | **Int, P** | **B (95% CI)** |
| Gross motor | 0.46 | -0.11 (-0.33; 0.11) | 0.048 | -0.12 (-0.29; 0.06) | 0.76 | -0.06 (-0.27; 0.14) | 0.045 | -0.13 (-0.32; 0.05) | 0.94 | -0.02 (-0.30; 0.25) |
|  |  | 0.01 (-0.23; 0.26) |  | 0.42 (-0.08; 0.92) |  | -0.01 (-0.27; 0.25) |  | 0.28 (-0.08; 0.65) |  | -0.06 (-0.93; 0.81) |
| Fine motor | 0.61 | -0.03 (-0.26; 0.19) | 0.67 | -0.08 (-0.26; 0.09) | 0.34 | -0.13 (-0.34; 0.08) | 0.17 | -0.13 (-0.31; 0.06) | 0.97 | -0.01 (-0.28; 0.25) |
|  |  | -0.12 (-0.37; 0.13) |  | 0.04 (-0.47; 0.54) |  | 0.04 (-0.23; 0.30) |  | 0.16 (-0.21; 0.53) |  | -0.03 (-0.87; 0.81) |
| Language | 0.58 | -0.04 (-0.26; 0.17) | 0.045 | -0.15 (-0.32; 0.02) | 0.34 | -0.01 (-0.21; 0.20) | 0.051 | -0.16 (-0.34; 0.02) | 0.34 | -0.17 (-0.42; 0.09) |
|  |  | -0.13 (-0.37; 0.11) |  | 0.39 (-0.10; 0.88) |  | -0.17 (-0.42; 0.09) |  | 0.24 (-0.12; 0.59) |  | -0.58 (-1.39; 0.23) |
| Social skills | 0.23 | -0.14 (-0.36; 0.08) | 0.03 | -0.12 (-0.29; 0.06) | 0.73 | -0.06 (-0.27; 0.15) | 0.77 | -0.05 (-0.23; 0.13) | 0.44 | 0.15 (-0.11; 0.41) |
|  |  | 0.07 (-0.18; 0.31) |  | 0.48 (-0.02; 0.99) |  | -0.003 (-0.26; 0.26) |  | 0.01 (-0.36; 0.38) |  | -0.19 (-1.02; 0.64) |
| Total score | 0.73 | -0.06 (-0.28; 0.15) | 0.02 | -0.15 (-0.32; 0.02) | 0.99 | -0.07 (-0.28; 0.13) | 0.01 | -0.19 (-0.37; -0.01) | 0.51 | -0.07 (-0.33; 0.18) |
|  |  | -0.12 (-0.36; 0.12) |  | 0.45 (-0.04; 0.94) |  | -0.07 (-0.33; 0.18) |  | 0.33 (-0.02; 0.69) |  | -0.36 (-1.16; 0.44) |
| Head circumference [1] | 0.02 | 0.15 (0.06; 0.24) | 0.18 | 0.09 (0.01; 0.16) | 0.49 | 0.05 (-0.04; 0.14) | 0.40 | 0.06 (-0.02; 0.14) | 0.38 | 0.08 (-0.03; 0.19) |
|  |  | -0.02 (-0.12; 0.08) |  | -0.07 (-0.28; 0.14) |  | 0.10 (-0.01; 0.21) |  | 0.13 (-0.02; 0.29) |  | 0.25 (-0.11; 0.61) |
| Data are p for interaction and stratum-specific estimates (95% confidence interval) based on linear mixed effect models adjusted for baseline value, age, sex, season, and site  Abbreviations; Int: Interaction, CI: confidence interval | | | | | | | | | | |

| **Table S.7:** **Per protocol analysis**; Effect of Milk protein and whey permeate in a lipid-based nutrient supplement (LNS) on child development among 662 children with stunting. Analysis based on the 2x2 factorial design among the 512 supplemented children and comparison of 512 supplemented vs 150 un-supplemented stunted children^1^ | | | | | | | |
| --- | --- | --- | --- | --- | --- | --- | --- |
|  | **Interaction,**  **p value** | **Milk vs soy protein**  **(n=259 vs n=253)** | | **Whey permeate vs maltodextrin**  **(n=257 vs n=255)** | | **LNS vs no supplement**  **(n=512 vs n=150)** | |
|  |  | **B (95% CI)** | **P value** | **B (95% CI)** | **P value** | **B (95% CI)** | **P value** |
| **MDAT domains** |  |  |  |  |  |  |  |
| Gross motor | 0.09 | -0.11 (-0.26; 0.05) | 0.18 | 0.07 (-0.08; 0.23) | 0.35 | -0.05 (-0.21; 0.12) | 0.58 |
| Fine motor | 0.97 | -0.06 (-0.22; 0.10) | 0.48 | -0.02 (-0.18; 0.14) | 0.81 | -0.07 (-0.24; 0.10) | 0.45 |
| Language | 0.75 | -0.03 (-0.19; 0.12) | 0.69 | -0.03 (-0.19; 0.13) | 0.73 | -0.09 (-0.25; 0.07) | 0.28 |
| Social skills | 0.26 | 0.001 (-0.15; 0.15) | 0.99 | 0.10 (-0.05; 0.25) | 0.20 | -0.05 (-0.21; 0.11) | 0.52 |
| Total score | 0.97 | -0.06 (-0.22; 0.09) | 0.41 | 0.001 (-0.15; 0.15) | 0.99 | -0.08 (-0.24; 0.08) | 0.31 |
| **Other outcomes** |  |  |  |  |  |  |  |
| Head circumference [1] | 0.06 | 0.03 (-0.04; 0.09) | 0.39 | -0.03 (-0.09; 0.03) | 0.37 | 0.08 (0.01; 0.15) | 0.02 |
| Data are p for interaction between milk protein and whey permeate, and main effect B (95% confidence interval) and p-value of each intervention based on linear mixed-effect models adjusted for baseline value of the outcome, age, sex, season, and site.  ^1^Only retained those children with good adherence that completed 12-week follow up period as reported elsewhere[2] | | | | | | | |

**References**

1. ICMJE ICoMJE. Recommendations for the Conduct, Reporting, Editing, and Publication of Scholarly Work in Medical Journals 2019 [cited 2021 5th May]. Available from: <http://www.icmje.org/icmje-recommendations.pdf>.

2. Joseph Mbabazi HP, Rolland Mutumba, Suzanne Filteau, Jack I. Lewis, Jonathan C. Wells, Mette F. Olsen, André Briend, Kim F. Michaelsen, Christian Mølgaard, Christian Ritz, Nicolette Nabukeera-Barungi, Ezekiel Mupere, Henrik Friis, Benedikte Grenov. Effect of milk protein and whey permeate in large quantity lipid-based nutrient supplement on linear growth and body composition among children with stunting: a randomized 2x2 factorial trial in Uganda. PLoS medicine. 2023;20(5):e1004227.
